# PPAR Ligands as Potential Modifiers of Breast Carcinoma Outcomes

**DOI:** 10.1155/2008/230893

**Published:** 2008-07-15

**Authors:** Ancha Baranova

**Affiliations:** Center for Biomedical Genomics and Bioinformatics, Molecular and Microbiology Department, College of Science, George Mason University, Fairfax, VA 22031, USA

## Abstract

Chemically synthesized ligands for nuclear receptors of the PPAR family modulate a number of physiological functions, particularly insulin resistance in the context of energy homeostasis and the metabolic syndrome. Additionally, these compounds may treat or prevent the development of many secondary consequences of the metabolic syndrome. Many PPAR agonists are also known to influence the proliferation and apoptosis of breast carcinoma cells though the experiments were carried out at suprapharmacological doses of PPAR ligands. It is possible that the breast epithelium of diabetics exposed to PPAR agonists will experience perturbation of the corresponding signaling pathway. Consequently, these patients' lifetime breast carcinoma risks could be modified, as their breast lesion incidence or the rates of the conversion of these lesions to carcinomas might vary upward or downward. PPAR activating treatment may also influence the progression of existing, undiagnosed invasive lesions. In this review, we attempt to summarize the possible influence of chemical PPAR ligands on the molecular pathways involved in the initiation and progression of breast carcinoma, with a major emphasis on PPAR*γ* agonists thiazolidinediones (TZDs).

## 1. INTRODUCTION

 Breast carcinoma is the most common nonskin cancer among women worldwide, responsible for about 375 000 deaths per year [1]. The probability of the development of breast carcinoma increases before menopause (ages 40–50) and then gradually decreases, possibly due to diminishing levels of circulating estrogens [2]. In developed countries, the prevalence of breast carcinoma is higher due to the frequency of known risk factors for the disease, including early age at menarche, nulliparity, late age at first birth, late menopause, and brief duration of breastfeeding [2]. All of these risk factors are tightly linked to hormonal background, particularly to lifelong exposure of breast tissue to endogenous estrogens [3]. Exogenous factors influencing breast carcinoma development include the use of oral contraceptives [4] and hormone replacement therapy [5, 6] as well as dietary or lifestyle-related variables. The latter category is rather vague, as it includes many factors detrimental to general health, such as high body-mass index [7], high fat intake [8], high red meat consumption [9], excessive alcohol consumption [10], and reduced physical activity [11].

A number of chemoprevention strategies for breast carcinoma are developed or under development. The noteworthy example is a tamoxifen chemoprevention in high-risk premenopausal women, which heralded the success of selective estrogen receptor modulators (SERMs) [12]. A new agent, raloxifene (Evista, Eli Lilly, IN, USA) also competes with endogenous estrogen for ER binding and shows similar promises with fewer side effects [13]. Interestingly, many potential breast carcinoma preventive agents studied earlier are also available over-the-counter and widely used by target populations. Examples of this kind include aspirin [14], soy isoflavones [15], and Vitamin D [16].

Recently, the universe of chemical compounds commonly encountered by current and future breast carcinoma patients has been enriched by a number of pharmacotherapeutic agents being prescribed as a lifelong support for common chronic diseases. Depending on the particular molecular pathways which these agents modulate, they may contribute to initial immortalization of breast epithelia, stimulate proliferation and invasion of existing tumor cells, or on the contrary, prevent the tumor’s development. For example, type II diabetes patients are routinely treated with chemically synthesized ligands for PPAR*γ*, thiazolidinedione (TZD), namely pioglitazone (Actos, Takeda/Lilly), and rosiglitazone (Avandia, GlaxoSmithKline). The glucose-lowering effects of these compounds are mediated primarily by decreasing insulin resistance and increasing glucose uptake by the skeletal muscles [17]. In addition, TZDs suppress glucose production in the liver [17]. These and other beneficial effects rapidly made TZDs a mainstream diabetes therapy [18].

In addition to their antidiabetic effects, TZDs are known to suppress the proliferation and induce apoptosis of breast carcinoma cells in vitro [19, 20]. It is likely that the breast epithelium of diabetics exposed to TZDs will also experience perturbation of the PPAR signaling pathway. Consequently, current or past TZD users’ lifetime breast carcinoma risks may be modified, as their breast lesion incidence or rates of the conversion of these lesions to carcinomas might change upward or downward. TZD treatment may also influence the progression of existing undiagnosed invasive lesions.

In addition to PPAR*γ* ligands, PPAR*α* [21] and PPAR*δ* [22] are currently being explored as potential cardiovascular therapeutics and metabolic syndrome alleviation agents. If these agents will be approved by FDA, it is very possible that in the next two or three decades the number of women exposed to one or another type of PPAR ligands may reach 10–15 million in the USA alone. Possible modifications of the breast carcinoma incidence and outcomes resulted by the chronic exposure to these compounds might translate into statistically significant changes visible in epidemiological survey data, similar to those seen in cohorts taking hormone replacement therapy [5, 6].

In this review, we attempt to summarize the possible influence of chemical PPAR ligands on the molecular pathways involved in the initiation and progression of breast carcinoma. Major emphasis will be on PPAR*γ*, as small molecular agonists of this nuclear receptor are widely used in the treatment of type II diabetes all over the world.

## 2. PPAR*γ* LIGANDS

A gene encoding nuclear hormone receptor, PPAR*γ*, expresses as two different mRNA isoforms derived from the alternative promoters, ubiquitous PPAR*γ*1 and adipose-specific PPAR*γ*2 [23]. Both isoforms stimulate adipogenesis; however, PPAR*γ*2 can be activated by lower concentrations of ligands [23]. Activated PPAR*γ* heterodimerizes with various coactivators [24, 25], which modulate the expression of genes with promoters containing bi-hexametric PPRE elements. These elements are widespread in the human genome, being present in both fatty acid metabolism and cell cycle control genes [26]. Moreover, the list of targets directly regulated by PPAR*γ* includes many genes which lack PPRE [27]. Most likely, this is due to either the binding of activated PPAR*γ* to other proteins that, in turn, serve as transcription factors (TFs) or the action of PPRE-containing genes providing delayed transcriptional response to PPAR*γ* ligation [27]. Knowledge about endogenous ligands for PPAR*γ* is limited. The list of these compounds includes polyunsaturated fatty acids (PUFAs) and eicosanoids, particularly lipoxygenase (LOX), and cyclooxygenase (COX) products [28]. An anti-inflammatory prostaglandin, 15-deoxy-D12,14-PGJ2 (15d-PGJ2), which is formed from PGD2 in vivo, is probably the most potent endogenous PPAR*γ* ligand [28]. Another powerful physiological stimulator of PPAR*γ* is oxidized phosphatidylcholine [29]. It should be mentioned that synthetic ligands of PPAR*γ* (TZDs) display stronger binding affinity to this nuclear receptor than its endogenous ligands, thus raising the question whether the list of natural PPAR*γ* ligands is complete.

### 2.1. Effects of the chronic exposure of the breast epithelium to PPAR*γ* agonists

PPAR*γ* is expressed in normal breast tissue and in many primary breast carcinoma specimens [30, 31]. Comparative studies of PPAR*γ* expression in breast carcinoma patients so far have produced contradictory results [32–34]. Described associations between *PPARG* polymorphisms and breast carcinoma are also discrepant: some researchers see a marginally significant increase in the risk of breast cancer among women homozygous for the Ala allele of PPAR*γ* (Pro12Ala), causing a reduction in the transcriptional activity of PPAR*γ*2 [35], while others stress that carriers of the same variant allele are at lower risk [36]. Since complete loss of PPAR*γ* signaling in clinical breast tumors seems to be a rare event [37], it is likely that patients undergoing chronic treatment with chemical ligands for PPAR*γ* will experience alteration in the behavior of both breast carcinoma cells and their normal counterparts. Patients with ER-positive tumors might benefit from TZD exposure more than those with ER-negative tumors, as the level of PPAR*γ* expression is significantly associated with the ER status of carcinoma cells [38].

Chemically synthesized ligands for PPAR*γ* (thiazolidinediones, or TZDs) have actively been used as insulin sensitizers since the late 90s [18]. In addition to their insulin resistance-alleviating effects, TZDs may influence an incidence or a progression of breast carcinoma lesions as they have been shown to suppress the proliferation rates of many types of cancer cells and induce either their differentiation or apoptosis in vitro [20, 39, 40]. Responsiveness to TZDs has been demonstrated for both normal human mammary epithelial cells [30] and breast cancer cells [41–43], although it was not uniformly seen in all experimental conditions [44]. TZDs suppress the cell cycle by repressing cyclins D1 and D3 [45], by stimulating expression of the tumor suppressor p53 and its effector p21 (WAF1/Cip1) [46], and by inhibiting the Akt/PTEN pathway [47]. Additionally, TZDs induce marked cellular acidosis in breast carcinoma cell lines, leading to a decrease in the number of viable cells [48]. Some effects of TZDs are independent of the transcriptional activities of PPAR*γ* [48]; these effects may be mediated through interactions of these compounds with other cellular targets.

The growth-suppressive properties of TZDs are complemented by their ability to induce apoptosis. Many breast tumors are naturally resistant to the apoptotic action of the tumor necrosis factor-related apoptosis-inducing ligand (TRAIL) and other similar agents. TZDs sensitize these cells to TRAIL [45], to anti-Fas IgM (CH11), and to tumor necrosis factor (TNF)-*α* [49]. It is tempting to speculate that TZDs might prevent the spread of microscopic breast tumors by sensitizing malignant cells to these endogenous apoptotic signals. Interestingly, TZDs also synergize with all-trans-retinoic acid (ATRA) to induce apoptosis in MCF-7 and primary breast carcinoma cells, but not in the normal breast epithelium [43]. Some TZDs also stimulate expression of apoptosis related genes, such as growth arrest and DNA damage-inducible gene 45 (*GADD45*) [50], *BRCA1* [51] and proline oxidase encoding gene *POX* [52]. In addition to intrinsic apoptotic pathways, TZDs are also capable of the direct stimulation of the *FASL* gene encoding Fas ligand that induces an apoptosis by cross-linking with the Fas receptor located on the membranes of the adjacent cells [53].

Additionally, TZDs block the invasion of tumor cells through upregulation of the tissue inhibitor of MMP-1/TIMP-1 and a subsequent decrease in MMP-9 gelatinolytic activities [54]. These observations have been supported by experiments with the murine mammary tumor cell line LMM3, which produces less metastatic nodules in lungs of animals treated by oral rosiglitazone [55]. It should be mentioned that the pronounced antitumor effects described above occur only at suprapharmacological doses of TZDs. It remains to be seen whether chronic exposure to TZDs could have therapeutic effects in patients with established breast tumors.

The effects described above are relevant only to some TZD users, namely, patients currently with breast tumors and those diagnosed with such tumors in the past. It is still unclear whether action of PPAR*γ* ligands is different within normal and tumor cells, and what would be effects of TXD exposure in cancer free individuals. There are some indications that PPAR*γ* ligands may influence the initial stages of breast carcinoma development, in particular, immortalization of the breast epithelia. One recent study demonstrated that exposure to low nontoxic doses of rosiglitazone (10 nM) reduces the frequency of spontaneous immortalization of Li-Fraumeni syndrome (LFS)-derived (p53 +/−, telomerase silent) breast epithelial cells by almost four times [56]. In these experimental settings, the antimutagenic properties of this widely prescribed TZD were superior to those of well-known chemopreventive agents such as sulindac sulfide and celecoxib [56]. It will be interesting to see whether exposure to TZD is capable of lowering the incidence of malignant foci in the breast epithelia genetically predisposed to breast carcinoma development, particularly that of carriers of mutations in *BRCA, BRCA2,* or *ATM*.

Some effects outlined above result from the interference of PPAR*γ* signaling with other pathways involved in breast carcinogenesis, particularly with estrogen receptor (ER)*α* and NF-*κ*B cascades. Agonists of PPAR*γ* may suppress NF-*κ*B dependent transcription either through an increase in physical interaction between PPAR*γ* and p65 [57] or through SUMOylation-dependent targeting of PPAR*γ* to NCoR/histone deacetylase-3 (HDAC3) corepressor complexes which prevent NCoR/HDAC3 clearance from NF-*κ*B target gene promoters [58]. The interplay between ER and PPAR*γ* signaling seems to be more complex. Many PPAR*γ* ligands, particularly troglitazone and ciglitazone, inhibit ER*α* signaling by stimulating proteasomal degradation of ER*α* [59].

On the other hand, one recent study’s findings are disturbing: in the breast cancer cell line MCF-7, commonly used as a model for ER-positive breast carcinoma, TZD rosiglitazone has been shown to induce both estrogen receptor response element activity and cell proliferation [44]. Even more disturbing is the fact that in dose-response assays higher concentrations of rosiglitazone inhibited proliferation, while lower concentrations of the same compound induced proliferation. Rosiglitazone-induced proliferation and ERE reporter activation were mediated by ER*α* and the extracellular signal-regulated kinase-mitogen activated protein kinase (ERK-MAPK) pathway [44]. The concentration-dependent nature of rosiglitazone’s effects may have tremendous clinical importance for the chronic users of TZDs. Moreover, these findings point at the possibility that the effects of the rosiglitazone might vary between individuals, as the bioavailability of rosiglitazone depends on the activity of the CYP2C9 and CYP2C8 enzymes [60], which are substantially polymorphic in human populations.

### 2.2. Chronic exposure to PPAR*γ* agonists influences nonepithelial cells participating in breast carcinoma development

In addition to the effects of PPAR*γ* ligands on premalignant and malignant breast epithelia, these compounds also produce profound changes in noncancerous cells. Some of these changes may be relevant to breast carcinoma outcomes. For example, PPAR*γ* ligands demonstrate antiangiogenic effects (reviewed in [40]), including direct suppression of the vascular endothelial growth factor (VEGF) and the angiopoietin-1 (Ang-1) gene transcription [61, 62]. On the other hand, in some noncancerous settings, PPAR*γ* ligands stimulate angiogenesis [63, 64], thus pointing to their involvement in remodeling tumor vessels rather than in suppressing angiogenesis per se.

In vitro experiments suggest that PPAR*γ* ligands act as differentiating agents in nonmalignant stromal cells. Malignant epithelialcells of breast tumors secrete growth factors and cytokines to prevent the differentiation of peri- and intratumoral stromal fibroblasts into mature adipocytes by downregulation of adipogenic factors such as the C/EBP*α* and PPAR*γ* [65]. In turn, underdifferentiated fibroblasts provide structural and secretory growth promoting support to tumor tissue [66]. Prolonged treatment with TZDs stimulates the differentiation of fibroblasts into adipocytes instead of myofibroblasts and interferes with transforming growth factor beta (TGF*β*) fibrogenic pathway, particularly, through attenuation of TGF*β*-driven type I collagen protein production [67]. Taken together, these effects of TZDs may to some degree counteract desmoplastic proliferative response promoted by tumor proximity and delay the formation of the scirrhous component of the breast tumors and the subsequent spread of tumor cells.

It must be taken into account that an interference of TZDs with TGF*β* signaling is a double-edged sword, since TGF*β* serves as both a tumor suppressor and a tumor promoter depending on tumor developmental stages and cellular context [19]. During the initial phase of breast tumorigenesis, the TGF*β* signal inhibits primary tumor development and growth by constraining cell division and possibly inducing apoptosis [68, 69]. In the later stages of breast carcinoma development, tumors lose their sensitivity to TGF*β*, but continue overproduction of the hormone. Excess TGF*β* acts upon stromal components of the tumor promoting the metastatic process through desmoplastic reaction, inhibiting host immune surveillance, and stimulating invasion and angiogenesis [70]. The outcome of the crosstalk between TGF*β* and PPAR*γ* in breast carcinoma patients should be dependent on stage of the particular breast lesion.

Last but not least, TZD therapy has been shown to produce an average weight gain of 4-5 kg, which cannot be explained by fluid retention [71]. The magnitude of weight gain correlates in part with improved metabolic control, that is, better responders are more prone to increases in body weight [72]. In turn, weight gain is associated with a significant increase in postmenopausal ER-positive/PR-positive breast cancer [73, 74]. It remains to be seen whether TZD-associated increases in adiposity contribute to breast carcinoma risks similarly to nonspecific weight gain.

### 2.3. Effects of TZDs on breast carcinogenesis in vivo

The PPAR*γ* agonist GW7845 delays the development of mammary tumors in immunocompetent mice treated with medroxyprogesterone acetate followed by DMBA administration by an average of 2 months [75]. In the classic rat model of mammary tumorigenesis employing nitrosomethylurea as a carcinogen, GW7845 also significantly reduces both tumor incidence and tumor weight [76]. Similarly, troglitazone, alone or in combination with RXR ligands, prevents the induction of preneoplastic lesions in a mouse mammary gland organ culture model treated by DMBA [77]. TZD treatment alone or in combination with ATRA suppresses tumor growth from breast carcinoma cells MCF-7 [43]. On the other hand, attempted rosiglitazone chemoprevention of breast carcinogenesis in the MMTV-HER-2/neu transgenic mouse model produced no encouraging data [78]. It is important to note that the mechanisms underlying various routes of the tumorigenesis in rodent breast differ substantially [79]; therefore, it is entirely possible that TZDs may modify outcomes only in some of the models studied. It is also possible that these effects might be either compound or dose-specific.

Recently, a few epidemiological studies have explored the association of TZD-based diabetes therapy and breast carcinoma incidence. The largest profiled cohort was the one covered by the Integrated Healthcare Information Services (IHCISs), Mass, USA, managed care database [80]. The relevant part of IHCIS allowed analysis of pharmacy and doctor’s office claim data related to 126 971 nonelderly USA diabetics with a mean followup time of 16.6 months. Importantly, each individual case of breast carcinoma (*N* = 513) was matched to up to five diabetes controls (cumulative *N* = 2557) using matched nested case-control design. The adjusted odds ratios and 95% CI for breast cancer from any exposure to TZD (mono- or combination therapy) compared to all non-TZD antidiabetic agents were 0.89 (0.68–1.15) [80]. Thus, neither a beneficial nor a deleterious effect of TZDs on the likelihood of breast carcinoma development was found. It should be mentioned that the median duration of followup in the studied cohort was rather short for the development of breast tumors. Studies following patients for longer periods of time are warranted.

Another group of researchers analyzed 1003 adult diabetic patients participating in a Vermont Diabetes Information System (VDIS) study and revealed a significant association between any cancer and the use of any TZD (OR = 1.59, 95% CI (1.03–2.44), *P* = .04) [79]. When TZDs were analyzed by compound, a significant association was found for rosiglitazone (OR = 1.89, 95% CI (1.11–3.19), *P* = .02), but not for pioglitazone. Stratification by gender showed a highly significant association between cancer prevalence and TZD use for women (OR = 2.07, 95% CI (1.18–3.63), *P* = .01) [81], but not for men. It is important to note that the number of the patients enrolled in this study is not allowed assessment of the risks for individual cancers. Nevertheless, the increase of tumor incidence in TZD using women points at the possible vulnerability of the breast epithelia.

Slightly more encouraging results were produced in the recently completed PROactive Study (PROspective pioglitAzone Clinical Trial In macroVascular Events). This study reviewed longitudinal data of 5238 diabetic patients treated with pioglitazone or with a placebo [82]. The incidence of breast carcinoma was nonsignificantly reduced in the pioglitazone-treated group (3 versus 11 cases in the equally sized pioglitazone and placebo arms of the study, resp.).

Several attempts to use TZDs as a means of therapy for breast carcinoma have been made so far. One trial of TZD as a monotherapy ended 5 months after it started, because troglitazone was withdrawn from the marker. This trial—performed in the cohort of patients with advanced breast cancer refractory to at least one chemotherapy regimen—resulted in no objective responses [83]. Another attempt at TZD monotherapy enrolled 38 women with early-stage lymph node negative breast carcinomas. This intervention was even shorter as rosiglitazone treatment (8 mg/d) was given between the time of diagnostic biopsy and definitive surgery. No significant effects on breast tumor cell proliferation were observed using Ki67 expression as an endpoint. Interestingly, rosiglitazone treatment leads to down-regulation of nuclear PPAR*γ* expression, as demonstrated by immunohistochemistry. Additionally, rosiglitazone intervention resulted in an increase of serum adiponectin concentrations (*P* < .001). Serum adiponectin negatively regulates breast cancer growth [84] and inhibits angiogenesis by suppression of endothelial cell proliferation and migration [85]. The potential therapeutic implications of rosiglitazone modulation of adiponectin levels require further study.

## 3. PPAR*α* LIGANDS

 The nuclear receptor PPAR*α* regulates lipid metabolism in general and *β*-oxidation of fatty acids in particular. Its gene, *PPARA*, expresses mainly in tissues with high energy requirements, particularly in the skeletal muscle, the heart, and the liver [86]. PPAR*α* is activated by a number of natural ligands, including various derivatives of fatty acids and leukotriene B4, and by common lipid-lowering drugs, particularly fenofibrate and gemfibrozil. Activated PPAR*α* exerts beneficial effects on lipid metabolism, raising cardioprotective high-density lipoprotein (HDL) cholesterol and lowering cardiovascular mortality [87]. In addition, activation of PPAR*α* may limit inflammation, both in the vessel endothelium and in other tissues as well as inhibit the fibrotic response. The apparent uniformly beneficial action of PPAR*α* agonists prompted the development of a number of these compounds. Among them, some exert dual affinity to PPAR*α* and PPAR*γ*. Dual agonists hold considerable promise in the management of insulin resistance, serving as major confounders for cardiovascular diseases and other comorbidities associated with metabolic syndrome.

Experimental data describing the effects of PPAR*α* agonists on tumor initiation and progression are limited. Long-term administration of PPAR*α* ligands clofibrate and WY-14643 in the rodent model induces hepatocellular neoplasms including adenomas and carcinomas [88]. PPAR*α* suppresses apoptosis in liver tissue in response to various peroxisome proliferator carcinogens, especially in the presence of TNF*α* [89]. As levels of TNF*α* are substantially elevated in obesity and in metabolic syndrome, it could be hypothesized that hepatocarcinogenesis may be an issue for long-term fibrate medicated patients. So far, epidemiological observations in fibrate treated populations have not produced any evidence that fibrates are associated with elevated risk of liver cancer or any other neoplasms in humans. As PPAR*α*-humanized mice are resistant to hepatocarcinogenic effects of fibrates, it seems that the response described in mouse models is species specific [90].

Studies of the nonhepatic tumorigenesis models indicate that in other tissues PPAR*α* agonists exert antiproliferative effects [91]. In the mouse model of skin carcinogenesis, an animal topically treated with PPAR*α* ligands exhibited an approximately 30% lower skin tumor yield compared with mice treated with vehicle, thus indicating that the activation of PPAR*α* may suppress the earliest stages of tumor development [92]. Additionally, PPAR*α* ligands possess strong antiangiogenic properties, as they suppress endothelial cell proliferation and VEGF production, upregulate TSP-1 and endostatin, and inhibit neovascularization [93, 94].

Studies concerning PPAR*α* activation in breast carcinomas are scarce. It is known that PPAR*α* is expressed and dynamically regulated in both ER-positive (MCF-7) and ER-negative (MDA-MB-231) human breast cancer cells. PPAR*α* activation significantly increases proliferation of both cell lines, and this increase is proportional to the endogenous level of PPAR*α* [95]. On the other hand, one recent study pointed at PPAR*α* as a possible contributor to the growth inhibitory effect of n-6 PUFA arachidonic acid exerted in the same pair of breast carcinomas cell lines [96].

PPAR*α* also reduces the sensitivity of MCF-7cells to histone deacetylase inhibitors [97]. Interestingly, there is an inverse relationship between mean PPAR*α* and ER*α* mRNA levels in ER-positive breast cancer cells [97]. These observations point to the possible involvement of PPAR*α* activation in mammary gland tumorigenesis and vouch for a longitudinal study of breast carcinoma incidence and progression in patients using fibrate therapy.

## 4. PPAR*δ* LIGANDS

The nuclear receptor PPAR*δ*, also known as PPAR*β*, is expressed ubiquitously. It controls a number of physiological functions, particularly cell proliferation and differentiation as well as inflammation and energy homeostasis [22]. Interestingly, PPAR*δ* is the only PPAR isoform that maintains repressor activity when bound to DNA. When unligated, PPAR*δ* can act as an intrinsic transcription repressor and inhibit the trans-activation activity of other PPARs [98]. It was suggested that PPAR*δ* serves as a gateway receptor capable of modulating PPAR*α* and PPAR*γ* activity [98]. The ligand binding pocket domain of PPAR*δ* is larger than that of other PPARs and is believed to accommodate the binding of various fatty acids and their derivatives [99]. A number of synthetic agonists are being developed for the same purpose with nanomolar affinities [100, 101], although none is currently marketed for clinical use in humans yet.

The physiological effects of activated PPAR*δ* have been studied extensively [22, 102]. The results of these studies suggest that sooner or later high-affinity PPAR*δ* synthetic drugs which uniquely target multiple components of the metabolic syndrome, including obesity, insulin resistance, hyperglycemia, dyslipidemia, and atherosclerosis will enter the market. Some of these compounds are already being subjected to phase I/II clinical trials. In light of this fact, it is important to establish experimental systems allowing rapid evaluation of the potential carcinogenic or chemopreventive effects of the synthetic PPAR*δ* ligands. Given that the prevalence of the metabolic syndrome and comorbidities associated with the disease is on the rise in both developed and developing countries, it is extremely important to watch for possible effects of anticipated chronic exposure to PPAR*δ* ligands upon common types of cancer, particularly upon breast carcinoma.

Alarmingly, PPAR*δ* selective agonists stimulate the growth of the hormone-dependent breast carcinoma cell lines T47D and MCF-7. In T47D cells, activation of PPAR*δ* stimulates expression of the proliferation marker Cdk2. In addition, an increase in the production of both VEGF and its receptor, FLT-1 has been noted, suggesting that PPAR*δ* may initiate an autocrine loop for cellular proliferation and possibly angiogenesis. Similar pro-proliferative effects of activated PPAR*δ* have been observed in endothelial cell cultures [103]. Further studies of angiogenic and growth-inducing properties of PPAR*δ* agonists in breast epithelia are warranted.

## 5. GENERAL REMARKS

**Figure 1:**
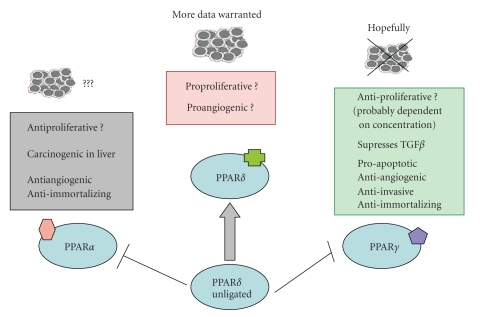
A summary of influence of PPAR ligands on the process of breast carcinogenesis.

It should be mentioned that breast carcinoma is not a single disease entity, but rather an extremely polymorphic spectrum of neoplastic pathologies which are fairly diverse in their molecular portraits. It is likely that both chemoprevention and treatment by PPAR ligands as well as their possible tumorigenic side effects will be selective to particular molecular subtypes of tumor, or will be relevant to certain stages of carcinoma progression (Figure 1). Therefore, much larger cohorts of patients followed for longer periods of time will have to be studied in order to reveal statistically significant modifications of the disease’s outcome. Chemoprevention studies of this type are prohibitively expensive, for example, the recently completed National Surgical Adjuvant Breast and Bowel Project Study of Tamoxifen and Raloxifene (STAR) trial with an endpoint of cancer incidence required the enrollment of 19 747 subjects from near 200 clinical centers throughout North America took 8 years before initial data analysis, and cost approximately $200 million [104, 105]. Before initiating large-scale efforts, a comparative study of the molecular portraits of breast carcinomas developed in chronic TZD users and in the general population needs to be completed. This kind of study could be performed using microarrays as a primary profiling means which should be complemented by validation efforts through the methods of immunohistochemistry, in situ hybridization of mRNA, and phosphoproteomics. The design of this study could be a challenge due to the difficulties with proper matching of groups compared and with eliminating common confounders. One of the possible ways to overcome this problem is to profile both malignant and normal breast epithelia samples of current TZD users to that of recently diagnosed diabetics never exposed to TZDs. Confirmed differences between the molecular portraits of tumors which initiated or progressed despite an exposure to PPAR ligand and subtype-matched tumors that arose on TZD free background may give some important clues to the design of a clinical trial aimed at chemoprevention-related endpoints.
